# Quarantine and Sanitary Convention

**Published:** 1859-07

**Authors:** 


Quarantine and Sanitary Convention.—This body held its third session in the City of New York, on the twenty-seventh of April, and the three following days. Dr. John H. Griscom, of New York, was elected President. The most remarkable feature in its proceedings was the vote, reached after considerable debate, declaring that, "in the absence of any evidence establishing the conclusion that yellow fever has ever been conveyed by one person to another, it is the opinion of this Convention that the personal quarantine of cases of yellow fever may be safely abolished, provided that femites of every kind be rigidly restricted." The resolution was carried by a vote of seventy yeas to four nays.

Of the reports of committees submitted to the Convention we shall be better prepared to speak when they are published in connection with an official account of the proceedings of the four days' meeting. The subjects embraced in them are of too important a character to be dismissed with a brief notice; and they will require from our hands a thorough analysis, even though they may not call for searching criticism. The labor this year has devolved chiefly on the medical delegates from Philadelphia. The greater part of the Report on Quarantine was written by Dr. Jewell, chairman of the committee, with the aid of Drs. La Roche and Condie. It consisted of five heads, viz.: the history of quarantine: have quarantines secured the object for which they were originally intended? If not, the reasons of their failure: what reforms are required to make quarantines more efficient and less burdensome: Is a uniform system of quarantine laws feasible? If so, to propose a plan by which the object may be accomplished: A consideration of the best means for purifying an infected vessel. The fourth question is discussed by Dr. Wragg; the fifth had been referred to Dr. Cleveland, but was not reported on. The other four members of the committee were Drs. John W. Moriarty, of Boston, Warner Cleveland, of Brooklyn, W. J. Wragg, of Charleston, S. C., and Wm. M. Kemp, of Baltimore. The Committee on Internal Hygiene, appointed by the Convention in Baltimore last year, was required to report under six different heads; each to be prepared by a member of the committee. The Report actually written and sent on to New York embraced four of these heads, viz.: 1. Upon the subject of disinfectants, their character, effects, and benefits, in connection with sanitary measures; by Dr. Van Bibber, of Baltimore. 2. Upon the importance of an ample supply of water, an adequate sewerage, and the proper disposal of the offal of cities; by Dr. J. H. Griscom, of New York. 3. Upon the importance and economy of sanitary measures to cities; by Dr. John Bell, of Philadelphia. 4. A draft of a sanitary code for cities; by Dr. Henry G. Clark, of Boston.

The reports were printed, by authority, before being presented to the Convention, and hence we can speak of their volume. The one on Quarantine takes up eighty-four pages octavo; that on the Internal Hygiene of Cities, introduced by a brief statement from the chairman, Dr. Miller, of Washington City, a hundred and ninety pages, of which Dr. Bell's report occupies a hundred and eighteen pages,* Dr. Van Bibber's thirty-eight, Dr. Griscom's five, and Dr. Clark's twenty-four pages. Dr. Griscom had not time to give more than a slight sketch of his subject, and hence Dr. Bell was left to occupy so much of the ground. The two subjects not reported on are, a Registration of Births, Marriages, and Deaths, assigned to Dr. Snow, of Providence; and on Vaccination as a Preventive of Variola, and on the value of Revaccination, assigned to Dr. Arnold, of Savannah.

New York, ever ready and liberal in her hospitalities, gave a dinner, complete in all the appliances of good cheer, to the members of the Quarantine and Sanitary Convention. The place of meeting next year will be at Boston, June 14th, 1860.

* If we are correctly informed, large additions will be made to this report by its author, in its passage through the press under the superintendence of the Publishing Committee, in New York.

PROFESSORIAL CHANGES.—The Faculty of the Pennsylvania Medical College, since the retirement of Dr. Stillé from the school, have resigned their professorships in a body, and have been succeeded by the appointment of the entire Faculty of the Philadelphia Medical College. This amalgamation has had the effect of making one school less in this city.

In the New York Medical College, Dr. Barker has resigned the Chair of Obstetrics, and Dr. Childs that of Anatomy. Their successors have not yet been appointed.

Dr. G. T. Elliot, Jr., has been made adjunct Professor of Obstetrics, and Dr. P. M. Markoe, adjunct Professor of Surgery, in the College of Physicians and Surgeons, of New York.

In the Savannah Medical College, Dr. W. R. Waring has been appointed to the Chair of Anatomy, made vacant by the resignation of Dr. Howard.

Dr. L. P. Yandell, who was connected with the University of Louisville since its foundation, has retired from the institution. We understand that Dr. David W. Yandell has been appointed to fill the vacancy.

Professor Wood has announced his intention of resigning the Chair of the Theory and Practice of Medicine in the University of Pennsylvania, at the close of the next session. Dr. Wood has filled this position with distinguished ability for a number of years, and his retirement from active professional life will prove a loss not only to the institution with which his name is so honorably connected, but to the profession throughout the whole country.

